# A Key Pre-Distribution Scheme Based on *µ*-PBIBD for Enhancing Resilience in Wireless Sensor Networks

**DOI:** 10.3390/s18051539

**Published:** 2018-05-12

**Authors:** Qi Yuan, Chunguang Ma, Haitao Yu, Xuefen Bian

**Affiliations:** 1 College of Computer Science and Technology, Harbin Engineering University, Harbin 150001, China; yuanqi@hrbeu.edu.cn; 2 College of Communication and Electronic Engineering, Qiqihar University, Qiqihar 161006, China; 3 College of Tourism, Guilin University of Technology, Guilin 541004, China; albertyht@glut.edu.cn; 4 College of Data Science and Technology, Heilongjiang University, Harbin 150080, China; bianxuefen@hlju.edu.cn

**Keywords:** wireless sensor networks, key pre-distribution, partially balanced incomplete block design, combinatorial design, resilience

## Abstract

Many key pre-distribution (KPD) schemes based on combinatorial design were proposed for secure communication of wireless sensor networks (WSNs). Due to complexity of constructing the combinatorial design, it is infeasible to generate key rings using the corresponding combinatorial design in large scale deployment of WSNs. In this paper, we present a definition of new combinatorial design, termed “*µ*-partially balanced incomplete block design (*µ*-PBIBD)”, which is a refinement of partially balanced incomplete block design (PBIBD), and then describe a 2-D construction of *µ*-PBIBD which is mapped to KPD in WSNs. Our approach is of simple construction which provides a strong key connectivity and a poor network resilience. To improve the network resilience of KPD based on 2-D *µ*-PBIBD, we propose a KPD scheme based on 3-D Ex-*µ*-PBIBD which is a construction of *µ*-PBIBD from 2-D space to 3-D space. Ex-*µ*-PBIBD KPD scheme improves network scalability and resilience while has better key connectivity. Theoretical analysis and comparison with the related schemes show that key pre-distribution scheme based on Ex-*µ*-PBIBD provides high network resilience and better key scalability, while it achieves a trade-off between network resilience and network connectivity.

## 1. Introduction

**Figure 1:**
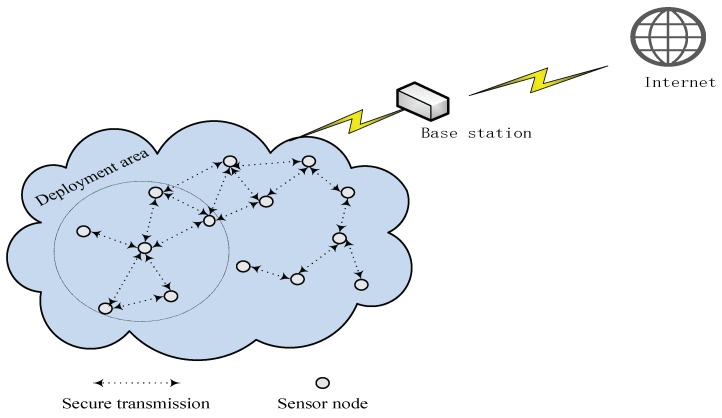
Environment of wireless sensor networks.

Wireless sensor networks have more and more extensive applications due to their properties in lower cost, low power consumption, easy deployment and self-organization [1,2]. Sensor nodes in wireless sensor networks are responsible for monitoring surrounding environment and transmitting the information on-request to base station in one-hop or multi-hop path. A general environment of wireless sensor networks is shown in Figure 1. When sensor networks are deployed in a hostile territory or a special region, they should secure the communication between two sensor nodes by encryption/decryption, safety authentication techniques and others [3,4,5,6,7,8]. Key management is a core of cryptographic system in WSNs, which is used to protect security in application of WSNs [9,10,11,12,13]. Although study on key management in WSNs becomes more mature, it still has a lot of challenges because of different required network size, wide application background, limited sensor performance and so on [14].

Key pre-distribution (KPD) scheme is one of the most extensive research directions of symmetric key management in WSNs [15,16,17]. A typical KPD scheme contains three phases: key pre-distribution, shared-key discovery and path-key establishment [13,18]. Key pre-distribution is an initialization phase, in which some keys selected from a large key pool are pre-distributed to each sensor to build a key ring. Shared-key discovery is to discover common pairwise keys between two nearby nodes by matching their key rings. In a path-key establishment phase, two nodes try to find one or more intermediary nodes that share common keys with them when the two neighboring nodes have no common pairwise keys. Various metrics of key pre-distribution scheme, such as network scalability, key connectivity, network resilience et al., are used for analyzing the merits and demerits of schemes in WSNs [19,20].

KPD schemes in WSNs are classified into probabilistic KPD scheme and deterministic KPD scheme based on the manner of key selection [6,12,16]. Typical probabilistic KPD schemes include random KPD, Q-composite KPD and polynomial pool based KPD [13]. Probabilistic KPD scheme randomly extracts a number of keys from key pool to form key rings of nodes, and its advantage is easy implementation due to its simple algorithm [21]. However, probabilistic KPD scheme only judges whether a pair of nodes have common keys by the mean of a probability value, and computers key connectivity by probabilistic result. Deterministic KPD scheme constructs key rings with a simple, straightforward model instead of selecting random key, which contributes to implementing shared-key discovery and path-key establishment. However, operations of these two phases, due to the absence of structure in key pre-distribution, are inherently complicated in randomized KDP scheme [20]. Meanwhile, performance metrics, such as scalability and connectivity, can be proven to be deterministic in a deterministic KPD scheme [22]. On the contrary, the deterministic value can not be obtained in a probabilistic scheme.

Combinatorial design theory is usually used for implementing deterministic KPD schemes. Due to the structural features of combinatorial design, metrics of combinatorial KPD scheme can easily be depicted. A general problem on existing combinatorial KPD schemes for WSNs is that construction of combinatorial designs mapped to KPD are complicated in implementation. Therefore, we focus on constructing a simpler combinatorial design applied to KPD scheme of WSNs, while performance metrics of KPD scheme should not be affected. A novel key pre-distribution scheme based on two-dimensional combinatorial design is introduced. Moreover, to enhance resilience and improve scalability, an extended three-dimensional combinatorial KPD scheme is proposed. The main contributions of our work are described as follows:

- A new combinatorial design (*µ*-PBIBD) is defined based on partially symmetric balanced incomplete block design.
- A *µ*-PBIBD is constructed in 2-D space, and a key pre-distribution scheme based on 2-D *µ*-PBIBD is proposed in which blocks are mapped to key rings. That is, shared-keys between nodes can be generated from common points between corresponding blocks. As a result, key connectivity of the proposed scheme depends on the construction of *µ*-PBIBD.
- To enhance network resilience of 2-D *µ*-PBIBD scheme, an Ex-*µ*-PBIBD is constructed by extending *µ*-PBIBD from 2-D space to 3-D space. Further, a key pre-distribution scheme based on 3-D Ex-*µ*-PBIBD is presented.
- Performance metrics of the proposed schemes are evaluated by theoretical analyses. Comparing with sBIBD scheme, RD and TD scheme, the results show that the proposed scheme has better scalability and higher resilience.

The remainder of this paper is organized as follows: In Section 2, related works on combinatorial design KPD schemes are introduced. Background knowledge of combinatorial design is described and a new combinatorial design is defined in Section 3. A *µ*-PBIBD is constructed and KPD scheme based on *µ*-PBIBD for WSNs is presented in Section 4. Then Section 5 proposes an extended *µ*-PBIBD based KPD scheme. Performance of the proposed scheme is analyzed and compared with the corresponding schemes in Section 6. Finally, the conclusions are drawn in Section 7.

## 2. Related Works

Combinatorial design theory is the part of combinatorial mathematics that deals with the existence and construction of systems of finite sets whose the existence have specified numerical properties [23]. Just because of these specified, easy-to-implement, numerical properties of combinatorial design theory, a series of studies on KPD scheme based on combinatorial design theory have been developed rapidly [24,25,26,27,28,29,30,31,32,33]. The first deterministic KPD scheme proposed by Comtepe and Yene [1] based on combinatorial design theory, which mapped Balanced Incomplete Block designs (BIBD) and Generalized Quadrangles (GQ) to KPD schemes, made key connectivity up to 1. Because of the difficulty of constructing BIBD and GQ, this KPD scheme supported only limited network size [9,30] and could not ensure keys pre-distribution according to actual demand about wireless sensor networks. Scheme [32] proposed a hybrid design according to complement of each block, i.e., when blocks of combinatorial design assigned to nodes were used up, a random subset of the complementary design blocks was distributed to the new-added nodes as key rings. This scheme supported larger-scale WSNs and improved the resilience of networks. Modiri et al. [30] introduced a new combinatorial design called residual design and mapped it to key pre-distribution scheme. This KPD scheme provided high connectivity while maintaining better scalability and resilience.

Stinson et al. [20,22,24,25] had been studying a series of combinatorial design based KPD since 2004. Lee and Stinson [20] introduced related knowledge of combinatorial set system to deterministic KPD schemes for WSNs. A strongly regular graph in [24] was used to product a network graph that represented whether two nodes share secret keys, and both one-way hash function and modified multi-space Bolm’ scheme were introduced to reduce efficiently storage overheads of keys and increase resilience. In schemes [25], Lee defined two basic types of combinatorial designs as “configurations” and “*µ*-common intersection design” and discussed their influence on the local connectivity and two-hop paths in WSNs. In schemes [20], Lee proposed a general framework to construct KPD schemes based on a transversal design (TD), and represented KPD schemes based on linear polynomials and quadratic polynomials. These schemes provided higher efficiency in a shared-key discovery phase with better connectivity and resiliency. Paterson and Stinson in [22] defined a general class of designs as “partially balanced *t*-designs”, which encompassed almost all of the proposed combinatorial designs used for KPD schemes. This general framework contributed to analyzing proposals of combinatorial KPD schemes and comparing with existing schemes, and easily evaluated which schemes possessed better performance metrics for a certain application. In [33], taking the problem with the restricted number of sensor nodes in combinatorial KPD into consideration, a universal method was proposed to compute metrics for connectivity and resilience of combinatorial KPD schemes. A deterministic method exploited a resolvable TD to adjust the network size by removing key rings and easily analyzed the properties of the scheme using the framework constructed in [22].

Taking into account the difficulty of implementation of scheme [32], Xia et al. [21] first constructed BIBD with Hadamard matrix, and then mapped it to a KPD scheme in WSNs. Furthermore, the network size of WSNs was doubled by complementary set design and the shared-key intensity was enhanced by key slicing. In [26], based on the divisible core pair-wise balanced design, key rings of nodes were constructed, where common blocks and particular blocks were mapped to key rings of common nodes and key rings of cluster head nodes, respectively. This scheme increased network scalability and had better resilience. Gao et al. [31] proposed a combinatorial design based KDP scheme for two-layer hierarchical WSNs. In this scheme, a key pre-distribution scheme was constructed with orthogonal array. A block associated with keys was assigned to a more capable node, and a random subset of a block associated with keys was allotted to a less capable node. This scheme obtained higher resilience and better tradeoff between performance metrics than some probabilistic schemes.

## 3. Preliminaries

Combinatorial design theory is the branch of combinatorics which focuses on designing subsets of a finite set to satisfy certain properties [23]. Block design is a type of combinatorial design. In the following section, a brief introduction of definitions and prerequisites of combinatorial design theory used in this paper are given.

### 3.1. Combinatorial Design

**Definition** **1** [34]**.** *Let V be a basic set of v elements (called points) with $V=\{p_{1},p_{2},\cdots ,p_{v}\}$ and B be a finite set of subsets (called blocks) of V. B is described as $B=\{B_{1},B_{2},\cdots ,B_{d}\}$ in which $B_{1},B_{2},\cdots ,B_{d}$ are d subsets of V. Then B is called “block design” of V.*

**Definition** **2** [32]**.** *If B is a block design of V that satisfies the following properties:*

*(1)*  Uniformity: Each block in B contains exactly k distinct points.
*(2)*  Regularity: Each point of V exists in exactly r different blocks of B.
*(3)*  Balance: Each pair of points of V exists in exactly λ blocks of B.

*B is called ”balanced incomplete block design (BIBD)” and denoted as B(v,d,r,k,λ). v,d,r,k,λ are parameters of the BIBD that satisfy dk=vr and λ(v−1)=r(k−1). In particular, when d = v and therefore r = k, a BIBD is called symmetric BIBD (sBIBD) which can be denoted as sB(v,k,λ).*

**Example** **1.** *Consider sB(v,k,λ)=(7,3,1) with V={1,2,3,4,5,6,7} and $B=\{B_{1},B_{2},\ldots ,B_{7}\}$. The blocks in B are: $B_{1}=\{1,2,3\}$, $B_{2}=\{1,4,5\}$, $B_{3}=\{1,6,7\}$, $B_{4}=\{2,4,6\}$, $B_{5}=\{2,5,7\}$, $B_{6}=\{3,4,7\}$, $B_{7}=\{3,5,6\}$.*

For every prime or prime power q≥2, there exists a $sB(q^{2}+q+1,q+1,1)$. Comtepe et al. [32] defined a mapping from $sB(q^{2}+q+1,q+1,1)$ to KPD and proposed a KPD scheme base on sBIBD. In this scheme, each point in *V* was associated with a distinct random key and each block was used as a key ring, providing the key pool having $v=q^{2}+q+1$ keys and $d=q^{2}+q+1$ key rings each having k=q+1 keys. In sBIBD, each pair of blocks intersected on one point and was mapped to KPD scheme in which each pair of key rings shared one key. As a result, the probability of key shared between each pair of nodes was always 1. When value of *q* was large, constructing $sB(q^{2}+q+1,q+1,1)$ was a NP-problem [32] which limited the size of sensor networks whose keys were pre-distributed. That is, this scheme was only theoretically feasible for a large scale of WSNs.

**Definition** **3** [20]**.** *A set system is a tripe (V,G,B), where V is a finite set of cardinality v, G is a partition of V into k parts (called groups) of size q and B is a block design of V with size k of blocks, which satisfies the following properties:*

*(1)*  |G∩B|=1, for every G∈G and every B∈B.
*(2)*  Every two points from different groups occurs in exactly λ blocks of B.

The tripe (V,G,B) is a transversal design of *V* which can be expressed as TD(λ,k,q). When λ=1, it can be written as TD(k,q). A TD(k,q) has the following properties: (1) There are exactly kq points and $q^{2}$ blocks; (2) every block contains exactly *k* points; and (3) every point occurs in exactly *q* blocks.

**Example** **2.** *Let*

*V* = {1, 2, 3, 4, 5, 6, 7, 8, 9, 10, 11, 12},

*G* = {{1, 2, 3}, {4, 5, 6}, {7, 8, 9}, {10, 11, 12}}, *and*

*B* = {{1, 4, 7, 10}, {1, 5, 8, 11}, {1, 6, 9, 12},   {2, 4, 8, 12}, {2, 5, 9, 10}, {2, 6, 7, 11},   {3, 4, 9, 11}, {3, 5, 7, 12}, {3, 6, 8, 10}}.

*Then (V,G,B) is a TD(4, 3) with a set V of |V| = kq = 12 points, G={G1,G2,⋯,Gk} of |G|= k = 4 groups and $B=\{B1,B2,\cdots ,Bq^{2}\}$ of |B|= q^2^ = 9 blocks.*

A TD(k,q), where *q* is a prime or a prime power, was constructed by Lee et al. in [20] as follows.

Let the point in *V* be denoted as (*a*, *b*), where a∈{0,1,⋯,k−1}, b∈Fq and 2≤k≤q. The construction of *V* is

$$\begin{array}{cc} V= & \{(0,0),(0,1),\cdots ,(0,q-1),\\ & (1,0),(1,1),\cdots ,(1,q-1),\\ & \cdots \\ & (k-1,0),(k-1,1),\cdots ,(k-1,q-1)\}. \end{array}$$

A group *G* of *V* is

$$\begin{array}{cc} G= & \{\{(0,0),(0,1),\cdots ,(0,q-1)\},\\ & \{(1,0),(1,1),\cdots ,(1,q-1)\},\\ & \cdots \\ & \{(k-1,0),(k-1,1),\cdots ,(k-1,q-1)\}\}. \end{array}$$

For every ordered pair (i,j)∈Fq×Fq, a block of *B* is defined as

$$B_{i,j}=\{(a,ia+j(\mathrm{mod}q))|0\leq a\leq k-1\}.$$

Then $B=\{B_{i,j}:(i,j)\in Fq\times Fq\}$. This tripe (V,G,B) is a TD(k,q).

Compared with sBIBD scheme proposed by Comtepe and Yener, this transversal design was simple in construction and corresponding KPD scheme was no limit to network size of WSNs.

### 3.2. µ-Partially Balanced Incomplete Block Design

A PBIBD is a generalization of a BIBD, in which each pair of points does not need to appear the same number of times [34]. The definition of PBIBD is given as follows:

**Definition** **4.** *If B is a block design of V that satisfies the following properties:*

*(1)*  *Uniformity: Each block in B contains exactly k distinct points.*
*(2)*  *Regularity: Each point of V exists in exactly r different blocks of B.*
*(3)*  *Partial Balance: Each pair of points of V exists in different numbers of blocks of B.*

*B is called “partial balanced incomplete block design (PBIBD)”. Further, we refine PBIBD to define a µ-PBIBD.*

**Definition** **5.** *Let F={λ1,λ2,⋯,λμ} be a set of positive integers. A µ-PBIBD is a pair (V, B), where V is a finite set of v elements (called “points”) and B is a set of d k-subsets (called “block”) of V, which satisfies the following properties:*

*(1)*  *(V, B) is regular, i.e., each point of V appears in exactly r different blocks of B.*
*(2)*  *(V, B) is uniform, i.e., the number of points in every block is k.*
*(3)*  *(V, B) is partial balance, i.e., every pair of points appears in λi blocks, for 1≤i≤μ.*

*The µ-PBIBD can be expressed as μ−PB(v,d,r,k,λ1,⋯,λμ), in which parameter r is called the degree of a point in V, k is called the rank of (V, B), and µ is called the class of (V, B).*

**Theorem** **1.** *μ−PB(v,d,r,k,λ1,⋯,λμ) exists only if dk = vr.*

**Theorem** **2.** *The number of common points in any two blocks is λi (1≤i≤μ). If µ = 1, a µ-PBIBD will degenerate into a BIBD, in which case any pair of points exists in λ1 blocks.*

In particular, when *d = v* and therefore *r = k*, a *µ*-PBIBD is called symmetric *µ*-PBIBD (*µ*-sPBIBD) which can be denoted as μ−sPB(v,k,λ1,⋯,λμ).

**Example** **3.** *Let V = {1, 2, 3, 4, 5, 6, 7, 8, 9, 10, 11, 12, 13, 14, 15} and $B=\{B_{1},B_{2},\cdots ,B_{15}\}$, where the blocks $B_{1},B_{2},\cdots ,B_{15}$ in B are:*

$$\begin{array}{l} B_{1}=\{2,3,4,5,6,11\};\;B_{2}=\{1,3,4,5,7,12\};\;B_{3}=\{1,2,4,5,8,13\};\;B_{4}=\{1,2,3,5,9,14\};\\ B_{5}=\{1,2,3,4,10,15\};\;B_{6}=\{1,7,8,9,10,11\};\;B_{7}=\{2,6,8,9,10,12\};\;B_{8}=\{3,6,7,9,10,13\};\\ B_{9}=\{4,6,7,8,10,14\};\;B_{10}=\{5,6,7,8,9,15\};\;B_{11}=\{1,6,12,13,14,15\};\;B_{12}=\{2,7,11,13,14,15\};\\ B_{13}=\{3,8,11,12,14,15\};\;B_{14}=\{4,9,11,12,13,15\};\;B_{15}=\{5,10,11,12,13,14\}. \end{array}$$

In this block design, there are 15 blocks and 15 points where each block contains 6 points and each point occurs in 6 blocks. Every pair of points appears in $\lambda_{1}$, $\lambda_{2}$ or $\lambda_{3}$ blocks, where $\lambda_{1}=1$, $\lambda_{2}=2$ and $\lambda_{3}=3$. Then the block design is a *µ*-sPBIBD which can be denoted as μ−sPB(15,6,1,2,3).

## 4. Key Pre-Distribution Based on *µ*-sPBIBD

In this section, we construct a basic sPBIBD and describe the mapping from *µ*-sPBIBD to KPD in WSNs.

### 4.1. A Construction of 2-D µ-sPBIBD

By combining with sB(v,k,λ) and TD(k,q) in Section 3.1, we use the representation of data elements in 2-D space to construct *µ*-$sPB(v,k,\lambda_{1},\cdots ,\lambda_{\mu })$ which can be described as follows.

Let points of *V* be expressed as (*a*, *b*), where (*a*, *b*) are coordinate of 2-D space elements for a∈{1,⋯,m} and b∈{1,⋯,n}. Then *V* is a set of cardinality mn, where

$$\begin{array}{cc} V= & \{(1,1),(1,2),\cdots ,(1,n),\\ & (2,1),(2,2),\cdots ,(2,n),\\ & \cdots \\ & (m,1),(m,2),\cdots ,(m,n)\}, \end{array}$$

for every ordered pair (a,b)∈{1,⋯,m}×{1,⋯,n}, a block in *V* is defined as

$$B_{a,b}=\{(i,b),(a,j)|1\leq i\leq m,i\neq a;\;1\leq j\leq n,j\neq b\}.$$

Let $B=\{B_{a,b}:(a,b)\in Z_{m}\times Z_{n}\}$.

The pair (*V*, *B*) has some following properties.

**Property** **1.** *In (V, B), V has mn points, B has exactly mn blocks, and the number of points in each block is exactly m + n − 2.*

**Proof.**  Constructed as before, *V* can be viewed as a 2-D space with the dimension m×n. Therefore, the number of points in *V* is mn; Each block $B_{a,b}$ in *B*, where $(a,b)\in Z_{m}\times Z_{n}$, is a set of coordinates of all elements on *a* row and *b* column except (*a*, *b*) in m×n 2-D space. Therefore, the number of blocks in *B* is mn and the number of points in each block is m−1+n−1=m+n−2. □

**Property** **2.** *In (V, B), every point in V occurs in exactly m+n−2 blocks.*

**Proof.**  According to the aforementioned construction of block, point (*a*, *b*) in *V* should appear in block $B_{a,\bar{b}}$ (where $\bar{b}$ is between 1 and *n* except *b*) and block $B_{\bar{a},b}$ (where $\bar{a}$ is between 1 and *m* except *a*). Therefore, the number of blocks containing point (*a*, *b*) is m+n−2. □

**Property** **3.** *In (V, B), there are three cases on the number λ of blocks in which any pair of points, say (a_1_, b_1_) and (a_2_, b_2_), is contained simultaneously. If $a_{1}\neq a_{2}$ and $b_{1}\neq b_{2}$, value of λ should be 2; If $a_{1}=a_{2}$ and $b_{1}\neq b_{2}$, value of λ should be n − 2; If $a_{1}\neq a_{2}$ and $b_{1}=b_{2}$, value of λ should be m − 2.*

**Proof.**  There are three cases on position relationship between two points in *V*. One is that, if points (*a*_1_, *b*_1_) and (*a*_2_, *b*_2_) in *V* lie on the different rows and columns, the two points should occur in blocks $B_{a_{1},b_{2}}$ and $B_{a_{2},b_{1}}$, and then λ=2. Another is that, if points (*a*_1_, *b*_1_) and (*a*_2_, *b*_2_) lie on the same row and different column, the two points should occur in exactly the blocks whose subscript are expressed by other points on the same row except these two points, and then λ=n−2. The third is that, if points (*a*_1_, *b*_1_) and (*a*_2_, *b*_2_) lie on the same column and different row, the two points should occur in exactly the blocks whose subscript are expressed by other points on the same column except these two points, and then λ=m−2.

Therefore, inferred from the three properties, (*V*, *B*) is *µ*-sPBIBD which can be denoted as μ−sPB(mn,m+n−2,2,m−2,n−2). □

### 4.2. 2-D µ-sPBIBD Based KDP Scheme

A key pool contains keys which will be selected in various ways to form key rings. These key rings need to be pre-distributed to sensor nodes before sensor nodes of WSNs are deployed. When nodes in WSNs transfer messages to their neighbor nodes, secure communications should be guaranteed by the common keys in key rings of communication nodes.

In KPD schemes based on 2-D *µ*-sPBIBD for WSNs with *M* sensor nodes, the mapping from 2-D *µ*-sPBIBD to KPD is described in Table 1. Each point in *V* can act as a key in the key pool and each block can be viewed as a key ring to distribute a sensor node, meaning that the number *d* of blocks should satisfy d≥M and if two blocks have common points, the two nodes which contain respectively the two blocks will have share-keys.

#### 4.2.1. Key Pre-Distribution Phase

In 2-D *µ*-sPBIBD scheme, keys in key pool are defined as the elements in 2-D space while the corresponding key IDs are expressed by coordinates of the elements in 2-D space. That is, points (1, 1), …, (1, *n*), …, (*a*, *b*), …, (*m*, 1), …, (*m*, *n*) in *V* are view as key IDs which are associated with keys in key pool. Point (*a*, *b*) and the corresponding key *key_a,b_* can be represented as a whole *P_a,b_*, where 1≤a≤m and 1≤b≤n, Then the key pool can be described as a set of *P_a,b_*. According to the construction of blocks proposed in Section 4.1, mn blocks $B_{a,b}$ are generated, where $(a,b)\in Z_{m}\times Z_{n}$, which can be denoted as $B_{a,b}=\{(P_{a,j},P_{i,b}|1\leq i\leq m,i\neq a;1\leq j\leq n,j\neq b\}$. The number of elements in block $B_{a,b}$ is *m* + *n* − 2. Elements *P_a,b_* in block $B_{a,b}$ are distributed as a key ring to a sensor node.

#### 4.2.2. Shared-Key Discovery Phase

When a sensor node needs to transmit the message to neighbor nodes, the node broadcasts its key IDs in key ring. The neighbors discover shared-keys with source node by comparing with their key IDs. Property 3 shows that there are three possibilities for the number of shared-keys between the two nodes: 2, *m* − 2 or *n* − 2.

Suppose that two sensor nodes N*_i_* and N*_j_* have *s* shared-keys, say ${key}_{1},{key}_{2},\cdots ,{key}_{s}$, where ${key}_{1},{key}_{2},\cdots ,{key}_{s}\in V$ and value of *s* is 2, *m* − 2 or *n* − 2, respectively. A session key between the two nodes can be generated from the shared-keys corresponding to common points between blocks. According to [20], a session key $K_{i,j}$ is established by a hash function *h*,

$$K_{i,j}=h(key_{1}||\cdots ||key_{s}||i||j)$$

This approach that computes session key by a hash function of common keys can improve the network resilience [6,20].

If two communication nodes fail to discover their shared-keys in the shared-key discovery phase, then path-key will be established. In 2-D *µ*-sPBIBD scheme, any pair of nodes can share at least two keys. Therefore, path-key establishment phase will not be considered.

### 4.3. 3-D Ex-µ-sPBIBD Based KPD Scheme 

In combinatorial KPD scheme, the more keys the blocks share, the more blocks are effected by a compromised block [32]. That is, network resilience contradicts with key connectivity [18]. Complete key connectivity inevitably leads to poor resilience in 2-D *µ*-sPBIBD based KPD scheme. In order to make a trade-off between resilience and connectivity, we propose an extended *µ*-PBIBD that can improve the resilience by reducing properly connectivity.

As mentioned in Section 4.1, a key pool can be viewed as 2-D space to store keys, in which key IDs are expressed by corresponding row-column coordinates of elements in 2-D space. In this subsection, we extend a key pool from 2-D space to 3-D space in which each key ID can be expressed by corresponding row-column-page coordinate of element in 3-D space. A extending *µ*-sPBIBD (Ex-*µ*-sPIBD) based KPD is proposed and KPD in 3-D space is described as follows.

Let *V* be a set of coordinates of q×q×q elements in 3-D space, which can be defined by

(1) $$\begin{array}{ll} \{ & \left[\begin{array}{l} \left(1,1,1\right),\cdots ,\left(1,b,1\right),\cdots ,\left(1,q,1\right)\\ \;\cdots \\ \left(a,1,1\right),\cdots ,\left(a,b,1\right),\cdots ,\left(a,q,1\right)\\ \;\cdots \\ \left(q,1,1\right),\cdots ,\left(q,b,1\right),\cdots ,\left(q,q,1\right) \end{array}\right]\\ & \cdots \\ & \left[\begin{array}{l} \left(1,1,c\right),\cdots ,\left(1,b,c\right),\cdots ,\left(1,q,c\right)\\ \;\cdots \\ \left(a,1,c\right),\cdots ,\left(a,b,c\right),\cdots ,\left(a,q,c\right)\\ \;\cdots \\ \left(q,1,c\right),\cdots ,\left(q,b,c\right),\cdots ,\left(q,q,c\right) \end{array}\right]\\ & \cdots \\ & \left[\begin{array}{l} \left(1,1,q\right),\cdots ,\left(1,b,q\right),\cdots ,\left(1,q,q\right)\\ \;\cdots \\ \left(a,1,q\right),\cdots ,\left(a,b,q\right),\cdots ,\left(a,q,q\right)\\ \;\cdots \\ \left(q,1,q\right),\cdots ,\left(q,b,q\right),\cdots ,\left(q,q,q\right) \end{array}\right]\} \end{array}$$

A point in set *V* is denoted as (*a*, *b*, *c*), where 1≤a,b,c≤q. The blocks in 3-D Ex-*µ*-sPBIBD are defined as

(2) $$B_{a,b,c}=\{(i,b,c),(a,j,c),(a,b,l)|1\leq i\leq q,i\neq a;\;1\leq j\leq q,j\neq b;1\leq l\leq q,l\neq c\},$$

where $(a,b,c)\in Z_{q}\times Z_{q}\times Z_{q}$.

Let

$$B=\{B_{a,b,c}:(a,b,c)\in Z_{q}\times Z_{q}\times Z_{q}\}.$$

In 3-D Ex-*µ*-sPBIBD, the number of blocks is $q^{3}$ and a block has 3*q* − 3 points. Mapping from Ex-*µ*-sPBIBD to KPD can be described in Table 2. 

A key pool is considered as 3-D space in which store q×q×q keys. Key IDs in the key pool are represented by row-column-page coordinate (*a*, *b*, *c*) of elements in 3-D space. A Key combining with the corresponding key ID is denoted as a whole $p_{a,b,c}$. A 3-D Ex-*µ*-sPBIBD is constructed by the approach similar to Section 4.1.

## 5. Theoretical Analysis

In this section, we analyze some important metrics of *µ*-sPBIBD based KPD scheme, such as connectivity, scalability and resilience.

### 5.1. Key Connectivity

Key connectivity is one of important metrics to evaluate the performance of KPD scheme in WSNs. Connectivity represents the ability of secure communication between nodes [26] and can be described by the probability that sensor nodes have shared-keys. If two nodes have no shared-keys, communication between them will use the third node to forward who has shared-keys with the two nodes, which will result in energy waste. Therefore, direct key connectivity can not only secure the networks but also save the communication overhead.

As noted in Section 4.2, KPD scheme based on 2-D *µ*-sPBIBD guarantees that any pair of key rings has $\lambda_{i}$ common keys, which means key connectivity of the proposed scheme can achieve 1. In the following, we study key connectivity of 3-D Ex-*µ*-sPBIBD scheme in WSNs.

**Figure 2:**
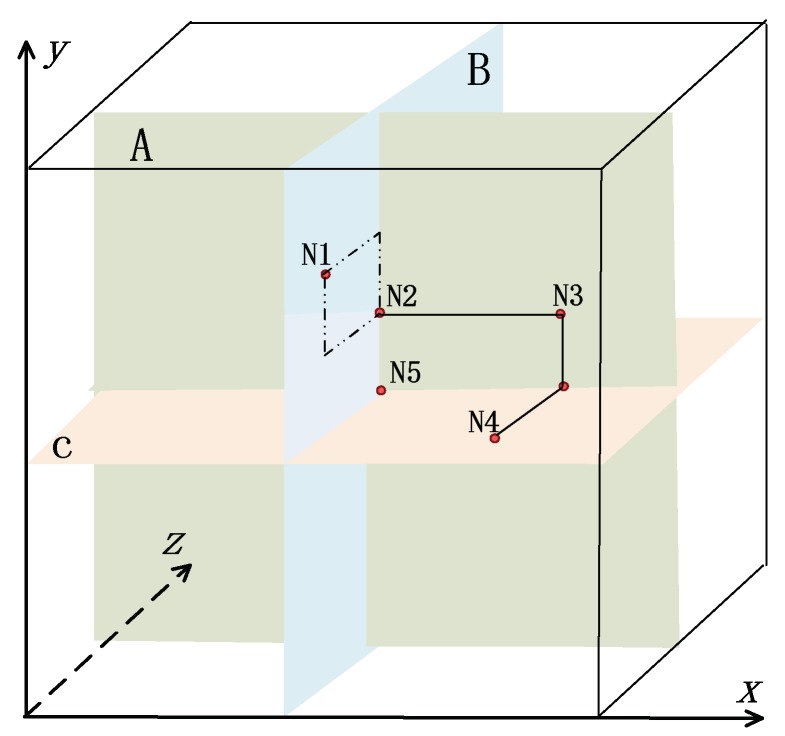
Relation among node, block and shared-key in 3-D Ex-*µ*-sPBIBD.

3-D space with dimension q×q×q is depicted in Figure 2. Taking N_1_ as example, the relation among node, block and 3-D space in 3-D Ex-*µ*-sPBIBD scheme are descripted as follow. Suppose that N_1_ is a sensor node in WSNs. Then a block $B_{a_{1},b_{1},c_{1}}$ constructed by Equation (2) is preloaded to N_1_ as a key ring. For simplicity, location of N_1_ in 3-D space is denoted as (*a*_1_, *b*_1_, *c*_1_).

If two nodes in 3-D space are coplanar, 3-D Ex-*µ*-sPBIBD will degenerate into 2-D *µ*-sPBIBD which has been described in Section 4.1. Therefore, two blocks have 2 or *q* − 2 common points, which means the two nodes have 2 or *q* − 2 shared-keys. If two nodes are preloaded non-coplanar blocks as key rings, they will have no shared-key and need to use path-key to secure communicate.

Let *V* be a set of |*V*| = *v* = 6×6×6 points and be expressed by Equation (1) where *q* = 6. According to Figure 2, nodes are denoted as N_1_, N_2_, N_3_, N_4_ and N_5_, while the corresponding blocks $B_{3,5,2}$, $B_{3,4,3}$, $B_{5,4,3}$, $B_{5,3,1}$ and $B_{3,3,3}$ can be described as follows.

$$\begin{array}{cc} B_{3,5,2}= & \{(1,5,2),(2,5,2),(4,5,2),(5,5,2),(6,5,2),\\ & \;(3,1,2),(3,2,2),(3,3,2),(3,4,2),(3,6,2),\\ & \;(3,5,1),(3,5,3),(3,5,4),(3,5,5),(3,5,6)\}\\ B_{3,4,3}= & \{(1,4,3),(2,4,3),(4,4,3),(5,4,3),(6,4,3),\\ & \;(3,1,3),(3,2,3),(3,3,3),(3,5,3),(3,6,3),\\ & \;(3,4,1),(3,4,2),(3,4,4),(3,4,5),(3,4,6)\}\\ B_{5,4,3}= & \{(1,4,3),(2,4,3),(3,4,3),(4,4,3),(6,4,3),\\ & \;(5,1,3),(5,2,3),(5,3,3),(5,5,3),(5,6,3),\\ & \;(5,4,1),(5,4,2),(5,4,4),(5,4,5),(5,4,6)\}\\ B_{5,3,1}= & \{(1,3,1),(2,3,1),(3,3,1),(4,3,1),(6,3,1),\\ & \;(5,1,1),(5,2,1),(5,4,1),(5,5,1),(5,6,1),\\ & \;(5,3,2),(5,3,3),(5,3,4),(5,3,5),(5,3,6)\}\\ B_{3,3,3}= & \{(1,3,3),(2,3,3),(4,3,3),(5,3,3),(6,3,3),\\ & \;(3,1,3),(3,2,3),(3,4,3),(3,5,3),(3,6,3),\\ & \;(3,3,1),(3,3,2),(3,3,4),(3,3,5),(3,3,6)\}. \end{array}$$

As shown in Figure 2, shared-keys between nodes have three cases. The first case is that, for example, blocks of N_1_ and N_2_ have two shared-keys, say (3, 5, 3) and (3, 4, 2), and the case are the same as N_1_ and N_5_, N_3_ and N_5_, N_3_ and N_4_, and N_4_ and N_5_. The second case is that blocks of nodes have *q* − 2 = 4 shared-keys. For example, shared-keys between N_2_ and N_3_ have (1, 4, 3), (2, 4, 3), (4, 4, 3) and (6, 4, 3). The third case is that blocks of nodes have no share-key in which we should establish their path-key.

Taking nodes N_1_ and N_3_ as example, we analyze the establishment of path-key between the two nodes. In Figure 2, N_2_ has shared-key with N_1_ and N_3_, and then a secure two-hop path between N_1_ and N_3_ (i.e., N_1_, N_2_, N_3_) is established.

Taking example for node N_5_ in Figure 2, we analyze the connectivity of Ex-*µ*-sPBIBD scheme. All nodes that are coplanar with N_5_ have the share-keys with N_5_. Therefore, the number of nodes on plane A, B and C that have share-keys with N_5_ is 3*q* (*q* − 1). The total number of nodes except N_5_ in WSNs is *q*^3^ − 1. Then direct connectivity of Ex-*µ*-sPBIBD is given by

(3) $$Con=\frac{3q(q-1)}{q^{3}-1}=\frac{3q}{q^{2}+q+1}.$$

Figure 2 illustrates shared relation of blocks and key connectivity of key rings. For simplicity, we replace block with node to illustrate key shared. There are three cases of key-shared between nodes: If two nodes, such as N_4_ and N_5_, lie on the same plane and have the different row and column subscript, the two nodes should have 2 shared-keys; If two nodes, such as N_2_ and N_3_, lie on the same plane and have the same row (or column subscript), the two nodes should share *q* − 2 keys; If two nodes, such as N_1_ and N_3_, are not coplanar, the two nodes should have no direct shared-key. 

### 5.2. Network Scalability

Network scalability reflects flexibility metrics of KPD scheme in WSNs and fails to effect security of network when new nodes join WSNs. Scalability can be expressed as the maximum number of nodes supported by KPD in WSNs. In the combinatorial KPD scheme, blocks are mapped to key rings. Therefore network scalability is equivalent to the number of blocks in combinatorial design.

In 2-D *µ*-sPBIBD, let the number of points in *V* be *v*, *v* can be decomposed into multiple forms as $m_{1}\times n_{1}$, $m_{2}\times n_{2},\cdots$. In terms of property 1, if *V* is described by 2-D spaces with different dimensions, the number of blocks of *µ*-sPBIBD will also be different which is $m_{1}+n_{1}-2$, $m_{2}+n_{2}-2,\cdots$, respectively. That is, scalability of KPD scheme based on 2-D *µ*-sPBIBD varies with the number of the corresponding key rings.

**Example** **4.** *Suppose the number of points in V is 10,000, 10,000 elements can be expressed in the form of 100 × 100, 50 × 200, 25 × 400, 20 × 500, 10 × 1000, 5 × 2000, 250 × 40 and 125 × 80, where the form 100 × 100 results in the minimum number of points in blocks in 2-D µ-sPBIBD.*

**Theorem** **3.** *Let v be expressed as q×q, $m_{1}\times n_{1}$, $m_{2}\times n_{2}\cdots$. In 2-D space, the form q×q corresponds to the minimum number of points in block.*

**Proof.**  Suppose that *v* can be described by two forms such as q×q and $\frac{q}{e}\times (q\times e)$, where $e,\frac{q}{e}\in Z^{+}$ and e≠1. In both cases, the number of points in blocks are 2*q* − 2 and $\frac{q}{e}+(q\times e)-2$, respectively. Comparing with the number of points in the two blocks, the result is as follow.

$$(2q-2)-(\frac{q}{e}+(q\times e)-2)=(-q)\frac{{\left(e-1\right)}^{2}}{e}<0$$

As described above, if *v* can be decomposed into many forms of multiplication of two numbers, the number of points of blocks will be the minimum in the case of *v* being expressed by a square of a certain number. That is, the corresponding 2-D space should hold the same row and column.

In 2-D *µ*-sPBIBD, the number of blocks is the same as the number of points in *V*. Therefore, the number of nodes in WSNs is also *v*. According to Theorem 3, in our proposed KPD scheme based on *µ*-sPBIBD, the number of keys in the key pool should be a minimum square of a number, which will lead to shorter key ring size under similar network scalability in WSNs. If the number of sensor nodes of WSNs is *n* and *n* = *q*^2^, the scalability of WSNs can be described as $\min\{q^{2}|q^{2}>n,\;q,n\in Z^{+}\}$.

In 3-D Ex-*µ*-sPBIBD, each point in *V* is denoted as coordinate of 3-D space which is the same as subscript of each block. As analyzed above, 3-D space should be defined as q×q×q, and then number of blocks in *V* is *q*^3^. That is, if the number of nodes in WSNs is *q*^3^, the scalability of WSNs can be described as $\min\{q^{3}|q^{3}>n,q,n\in Z^{+}\}$. □

### 5.3. Network Resilience

Resilience represents security metrics of KPD against node capture in WSN. Because low performance nodes in WSNs are not equipped with tamper-resistant hardware [35] once one node is captured by an adversary, all of the information stored in the node including key material will be exposed. The adversary may use the captured keys to decrypt communication between other nodes that using the same keys. When the number of compromised sensor nodes reaches a certain value, all keys in the key pool will be exposed and the whole WSNs will be collapsed.

Resilience reflects the extent that the compromised nodes affect the remaining non-compromised nodes when WSNs suffer from attack of node capture. Resilience of WSNs is expressed as *Res*(*x*), which denotes the broken probability of a link between two fixed non-compromised nodes when an attacker captures *x* other nodes [20]. The lower the value of *Res*(*x*) is, the stronger the resilience of WSNs will be.

#### 5.3.1. Resilience of 2-D *µ*-sPBIBD

**Figure 3:**
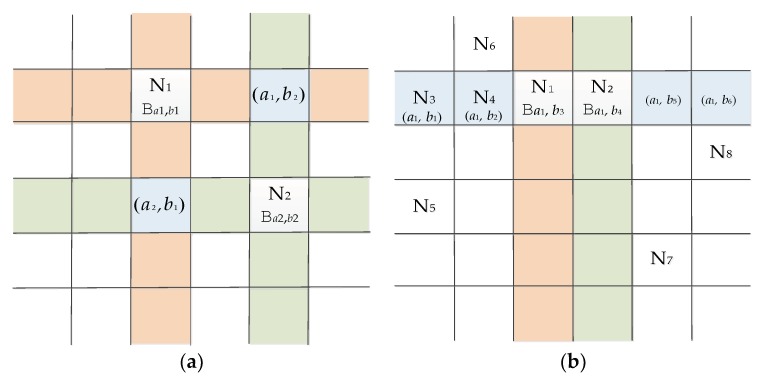
Key distribution and key-shared corresponding to 2-D sPBIBD. (**a**) 2 shared-keys; (**b**) *q* − 2 shared-keys.

As noted in Section 5.2, let *V* be square of *q* in 2-D *µ*-sPBIBD. Then two nodes have 2 or *q* − 2 shared-keys. In Figure 3, 2-D space with dimension q×q is depicted. Taking N_1_ in Figure 3a as example, the relation among node, block and 2-D space in 2-D *µ*-sPBIBD scheme is descripted as follows. Suppose that N_1_ is a sensor node in WSNs, a block $B_{a_{1},b_{1}}$ constructed in Section 4.1 is preloaded to N_1_ as a key ring in which (*a*_1_, *b*_1_) is a point of *V*. Then, for simplicity, location of N_1_ in 2-D space is denoted as (*a*_1_, *b*_1_).

1. If the number of shared-keys is 2

Suppose that node N_1_ and N_2_ share two keys. Two blocks corresponding to key rings preloaded to N_1_ and N_2_ are denoted as $B_{a_{1},b_{1}}$ and $B_{a_{2},b_{2}}$. As presented in Figure 3, in 2-D space, points in $B_{a_{1},b_{1}}$ cover orange and blue segments, while points in $B_{a_{2},b_{2}}$ cover green and blue segments. Figure 3a illustrates that $B_{a_{1},b_{1}}$ and $B_{a_{2},b_{2}}$ have common points (*a*_1_, *b*_2_) and (*a_2_, b*_1_) which represent key ID of two shared-keys between N_1_ and N_2_ (for simplicity, in the following analyses, we replace key with key ID).

Resilience is repressed by the probability that communication between N_1_ and N_2_ will be compromised after *x* random nodes are captured. Suppose that $H_{a_{1},b_{2}}$ and $H_{a_{2},b_{1}}$ are two sets of blocks including (*a*_1_, *b*_2_) and (*a*_2_, *b*_1_), respectively. From Property 3, we have that

$$|H_{a_{1},b_{1}}|=|H_{a_{2},b_{2}}|=2q-2$$

and $|H_{a_{1},b_{1}}\cap H_{a_{2},b_{2}}|=2$. Then

$$|H_{a_{1},b_{1}}\cup H_{a_{2},b_{2}}|=2\left(2q-2\right)-2=4q-6.$$

To secure the communication between N_1_ and N_2_, (*a*_1_, *b*_2_) or (*a*_2_, *b*_1_) should not exist in the blocks associated with the *x* captured nodes. The number of ways of choosing *x* nodes unrelated to (*a*_1_, *b*_2_) is $\left(\begin{array}{c} q^{2}-2q+2\\ x \end{array}\right)$. Similarly, the number of ways of choosing *x* nodes unrelated to (*a*_2_, *b*_1_) is $\left(\begin{array}{c} q^{2}-2q+2\\ x \end{array}\right)$. Then the number of ways of choosing *x* nodes unrelated to $\left|H_{a_{1},b_{1}}\cup H_{a_{2},b_{2}}\right|$ is $\left(\begin{array}{c} q^{2}-4q+6\\ x \end{array}\right)$. Therefore, if *x* nodes are captured, network resilience, which is represented by the probability that communication with two fixed nodes is broken, can be given by

(4) $$\begin{array}{cc} Res_{1}(x)= & 1-\frac{2\left(\begin{array}{c} q^{2}-2q+2\\ x \end{array}\right)-\left(\begin{array}{c} q^{2}-4q+6\\ x \end{array}\right)}{\left(\begin{array}{c} q^{2}-2\\ x \end{array}\right)}\\ & \approx 1-2{\left(1-\frac{2q-4}{q^{2}-2}\right)}^{x}+{\left(1-\frac{4q-8}{q^{2}-2}\right)}^{x} \end{array}$$

2. If the number of shared-key is *q* − 2

Two blocks will share *q* − 2 keys if the two blocks corresponding to two key rings in node N_1_ and N_2_ have the same row-subscript (or column-subscript). In Figure 3b, blocks $B_{a_{1},b_{3}}$ and $B_{a_{1},b_{4}}$ have the same row-subscript. Then the common points between $B_{a_{1},b_{3}}$ and $B_{a_{1},b_{4}}$ are all elements in *a*_1_ row except (*a*_1_, *b*_3_) and (*a*_1_, *b*_4_).

As illustrated in Figure 3b, suppose blocks in N_1_ and N_2_ have the same row (or column). If an attacker captures *x* nodes, N_1_ and N_2_ will compromise in the following three cases:

(1) In *x* captured nodes, there are at last two nodes, such as N_3_ and N_4_, that the corresponding blocks have the same row (or column) subscript as the blocks in N_1_ and N_2_.
(2) In *x* captured nodes, there are one node, such as N_3_, that the corresponding block has the same row (or column) subscript as the blocks in N_1_ and N_2_, and then another node, such as N_5_, must be the node that corresponding block has the same column (or row) subscript as block in N_3_.
(3) In *x* captured nodes, if subscripts of blocks in *x* captured nodes are different with those of N_1_ and N_2_, *x* should be greater than or equal to *q* − 2 and there are at least *q* − 2 captured nodes that column (or row) subscripts of the corresponding blocks are different with those of N_1_ and N_2_. Meanwhile, column (or row) subscripts of corresponding *q* − 2 blocks are different from each other. For example, in Figure 3b, N_5_, N_6_, N_7_ and N_8_ are four nodes of *x* compromised nodes.

Resilience of the first two cases will be given by

(5) $$Res_{2\_1}(x)=1-\frac{\left(\begin{array}{c} q^{2}-q\\ x \end{array}\right)+\left(\begin{array}{c} q-2\\ 1 \end{array}\right)\left(\begin{array}{c} q^{2}-q\\ x-1 \end{array}\right)}{\left(\begin{array}{c} q^{2}-2\\ x \end{array}\right)}.$$

In the third case, the number of ways of choosing *x* compromised nodes is given by

(6) $$\begin{array}{cc} Ch(x)= & \left(\begin{array}{c} q(q-1)\\ x \end{array}\right)\\ & -\left(\begin{array}{c} q-2\\ 1 \end{array}\right)\left(\begin{array}{c} \left(q-1)(q-1\right)\\ x \end{array}\right)\\ & +\left(\begin{array}{c} q-2\\ 2 \end{array}\right)\left(\begin{array}{c} \left(q-1)(q-2\right)\\ x \end{array}\right)\\ & +\\ & \cdots \\ & {+(-1)}^{\theta }\left(\begin{array}{c} q-2\\ \theta \end{array}\right)\left(\begin{array}{c} \left(q-1)(q-\theta \right)\\ x \end{array}\right)\\ & +\cdots \\ & \;(q-1)(q-2)\geq x\geq q-2 \end{array}$$

and resilience of the third case will be given by

(7) $$Res_{2\_2}(x)=\frac{Ch(x)}{\left(\begin{array}{c} q(q-1)\\ x \end{array}\right)}$$

Then, if two nodes have *q* − 2 shared-keys, resilience can be written as:

(8) $$Res_{2}(x)=Res_{2\_1}(x)+Res_{2\_2}(x)$$

In terms of the construction of *µ*-sPBIBD, the probability that two blocks share *q* − 2 points is given by

(9) $$pro_{2}=\frac{2q-2}{q^{2}-1}=\frac{2}{q+1}.$$

The probability that two blocks share 2 points is given by

(10) $$pro_{1}=1-pro_{2}=\frac{q-1}{q+1}.$$

Finally, resilience of KPD scheme based on *µ*-sPBIBD can be computed by Equations (4) and (8)–(10). The resilience is expressed as follows:

(11) $$\begin{array}{cc} Res(x) & =pro_{1}\times Res_{1}(x)+pro_{2}\times Res_{2}(x)\\ & =\frac{q-1}{q+1}\left(1-2{\left(1-\frac{2q-4}{q^{2}-2}\right)}^{x}+{\left(1-\frac{4q-8}{q^{2}-2}\right)}^{x}\right)\\ & +\frac{2}{q+1}(1-\frac{\left(\begin{array}{c} q^{2}-q\\ x \end{array}\right)+\left(\begin{array}{c} q-2\\ 1 \end{array}\right)\left(\begin{array}{c} q^{2}-q\\ x-1 \end{array}\right)}{\left(\begin{array}{c} q^{2}-2\\ x \end{array}\right)}+\frac{Ch(x)}{\left(\begin{array}{c} q(q-1)\\ x \end{array}\right)}) \end{array}$$

#### 5.3.2. Resilience of 3-D Ex-*µ*-sPBIBD

Resilience of 3-D Ex-*µ*-sPBIBD are similar to 2-D *µ*-sPBIBD. Suppose that *x* random nodes are captured. Resilience can be analyzed as follows.

1. If the number of shared-keys is 2

Suppose that subscripts of blocks of two nodes have different row and column in the same plane. The two nodes have two shared-keys, say (*a*_1_, *b*_1_, *c*_1_) and (*a*_2_, *b*_2_, *c*_2_). For example, suppose N_1_ and N_2_ in Figure 2 have two shared-keys and the corresponding points are (3, 5, 3) and (3, 4, 2). $H_{a_{1},b_{1},c_{1}}$ and $H_{a_{2},b_{2},c_{2}}$ are sets of blocks containing (*a*_1_, *b*_1_, *c*_1_) and (*a*_2_, *b*_2_, *c*_2_), respectively, where *a*_1_ = *a*_2_, $b_{1}\neq b_{2}$ and $c_{1}\neq c_{2}$. According to Property 3, we have

$$|H_{a_{1},b_{1},c_{1}}|=|H_{a_{2},b_{2},c_{2}}|=3q-3$$

and

$$|H_{a_{1},b_{1},c_{1}}\cap H_{a_{2},b_{2},c_{2}}|=2.$$

Then

$$|H_{a_{1},b_{1},c_{1}}\cup H_{a_{2},b_{2},c_{2}}|=2\left(3q-3\right)-2=6q-8.$$

In order to ensure the security of a link between N_1_ and N_2_, key rings of *x* captured nodes fail to contain the two keys (*a*_1_, *b*_1_, *c*_1_) and (*a*_2_, *b*_2_, *c*_2_). The number of ways of choosing *x* nodes unrelated to (*a*_1_, *b*_1_, *c*_1_) is $\left(\begin{array}{c} q^{3}-3q+3\\ x \end{array}\right)$. Similarly, the number of ways of choosing *x* nodes unrelated to (*a*_2_, *b*_2_, *c*_2_) is $\left(\begin{array}{c} q^{3}-3q+3\\ x \end{array}\right)$. Then the number of ways of choosing *x* nodes unrelated to $\left|H_{a_{1},b_{1},c_{1}}\cup H_{a_{2},b_{2},c_{2}}\right|$ is $\left(\begin{array}{c} q^{3}-6q+8\\ x \end{array}\right)$. Therefore, if *x* nodes are captured, the probability $Res_{1}^{\prime }(x)$ which a link between the two fixed nodes is broken will be given as follows:

(12) $$\begin{array}{cc} Res_{1}^{\prime }(x) & =1-\frac{2\left(\begin{array}{c} q^{3}-3q+3\\ x \end{array}\right)-\left(\begin{array}{c} q^{3}-6q+8\\ x \end{array}\right)}{\left(\begin{array}{c} q^{3}-2\\ x \end{array}\right)}\\ & \approx 1-2{\left(1-\frac{3q-5}{q^{3}-2}\right)}^{x}+{\left(1-\frac{6q-10}{q^{3}-2}\right)}^{x}. \end{array}$$

2. If the number of shared-keys is *q* − 2

If subscripts of two blocks are coplanar and with the same row (or column), their corresponding nodes will have *q* − 2 shared-keys. Taking N_2_ and N_3_ as example in Figure 2, we compute network resilience.

Coplanar two blocks in 3-D Ex-*µ*-sPBIBD can be viewed as two blocks in 2-D *µ*-sPBIBD. For simplicity, as analyzed in Section 5.3.1, it is similar to Equations (4)–(7) that the resilience of this case can be given by

(13) $$Res_{2}^{\prime }(x)=1-\frac{\left(\begin{array}{c} q^{2}-q\\ x \end{array}\right)+\left(\begin{array}{c} q-2\\ 1 \end{array}\right)\left(\begin{array}{c} q^{2}-q\\ x-1 \end{array}\right)}{\left(\begin{array}{c} q^{3}-2\\ x \end{array}\right)}+\frac{Ch(x)}{\left(\begin{array}{c} q(q-1)\\ x \end{array}\right)}$$

In terms of construction of 3-D Ex-*µ*-sPBIBD, the probability that two blocks share 2 points can be given by

(14) $$pro_{1}^{\prime }=\frac{3q-3}{q^{2}+q+1}.$$

The probability that two blocks share *q* − 2 points can be given by

(15) $$pro_{2}^{\prime }=\frac{3q-3}{q^{3}-1}=\frac{3}{q^{2}+q+1}$$

Finally, resiliency of KPD scheme based on 3-D Ex-*µ*-sPBIBD can be computed by Equations (12)–(15). Resilience can be expressed as follows,

(16) $$\begin{array}{cc} Res^{\prime }(x) & =pro_{1}^{\prime }\times Res_{1}^{\prime }(x)+pro_{2}^{\prime }\times Res_{2}^{\prime }(x)\\ & =\frac{3q-3}{q^{2}+q+1}\left(1-2{\left(1-\frac{3q-5}{q^{3}-2}\right)}^{x}+{\left(1-\frac{6q-10}{q^{3}-2}\right)}^{x}\right)\\ & +\frac{3}{q^{2}+q+1}(1-\frac{\left(\begin{array}{c} q^{2}-q\\ x \end{array}\right)+\left(\begin{array}{c} q-2\\ 1 \end{array}\right)\left(\begin{array}{c} q^{2}-q\\ x-1 \end{array}\right)}{\left(\begin{array}{c} q^{3}-2\\ x \end{array}\right)}+\frac{Ch(x)}{\left(\begin{array}{c} q(q-1)\\ x \end{array}\right)}) \end{array}$$

## 6. Performance Comparison

In order to better analyze the performance of the proposed method, we compare with other combinatorial design based KPD schemes. Symmetric BIBD scheme [32] is a classical combinatorial design based deterministic key pre-distribution scheme, which mapped a symmetric design with parameters (*q*^2^ + *q* + 1, *q* + 1, 1) to KPD scheme. RD scheme [30] constructed a residual design (RD) based on sBIBD with parameters (*q*^2^ + *q* + 1, *q* + 1, 1) and was first time that used RD to KPD scheme, which improved the resilience and scalability comparing with sBIBD scheme. TD scheme [20] employed linear construction and quadratic construction of transversal designs which were expressed as TD(k,q) and TD(λ,k,q), respectively, and it offered a lot of flexibility in trading off the various metrics.

In this section, we compare the proposed schemes with sBIBD scheme, RD scheme and linear TD scheme according to different criteria. For the sake of clarity, the parameters of different KPD schemes are listed in Table 3. We can find that metrics of linear TD scheme depend on two parameters *k* and *q*, which is different from others combinatorial schemes that only depend on one parameter.

### 6.1. Network Scalability

According to Table 3, we can obtain network scalability of these schemes. In sBIBD scheme, the key ring size was *k = q* + 1 and the maximum size of network supported by sBIBD scheme was *q*^2^ + *q* + 1. In RD scheme, the key ring size was *k = q* and the scalability of RD scheme was computed as (*q*^2^ + *q* + 1)(*q* + 1). In linear TD scheme, the key ring size was *k* and the probability that two sensor nodes shared a common key was *Pr*_1_. Then a prime *q* was chosen such that *q* + 1 ≤ *k*/*Pr*_1_, and the maximum scale of network supported by linear TD scheme was *q*^2^ [20]. In 2-D *µ*-PBIBD scheme, each node is preloaded with *k* = 2*q* − 2 distinct keys and the maximum network size that can be supported by 2-D *µ*-PBIBD scheme is *q*^2^. The key ring size is *k =* 3*q* − 3 in 3-D Ex-*µ*-PBIBD scheme which can support network size up to *q*^3^.

**Figure 4:**
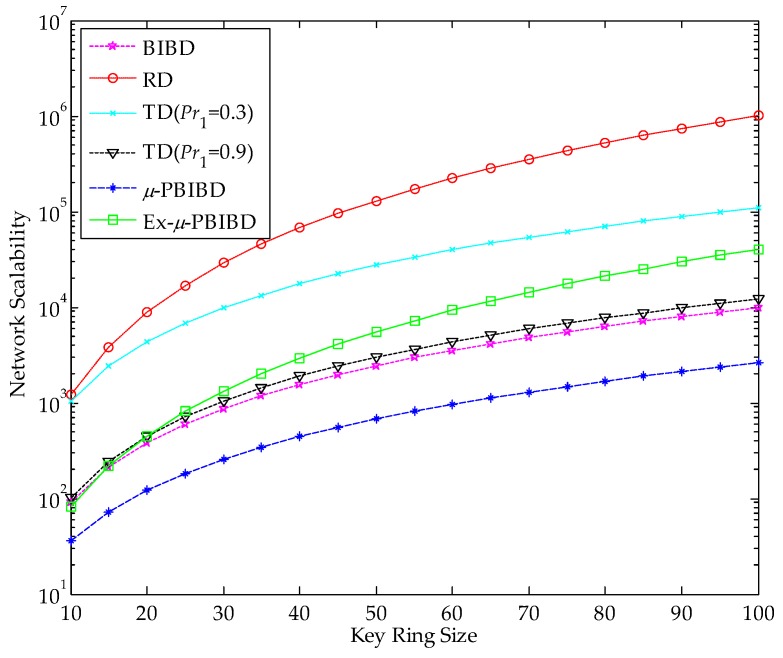
Comparison of network scalability of different KPD schemes at the same key ring size *k*.

The scalability of *µ*-PBIBD and Ex-*µ*-PBIBD are compared with sBIBD, RD and TD schemes when size of key ring increases from 10 to 100 by increments of 10. For linear TD scheme, we analyze the scalability in the case of *Pr*_1_ = 0.3 and *Pr*_1_ = 0.9. As expected, Ex-*µ*-PBIBD scheme performs better network scalability than *µ*-PBIBD scheme. Figure 4 shows that at the same key ring size, scalability of Ex-PBIBD is higher than, PBIBD, BIBD and TD(*Pr*_1_ = 0.9) scheme, while it is lower than RD and TD(*Pr*_1_ = 0.3) scheme. When key ring size is up to 100, the network sizes of the schemes in Figure 4 are 1020201, 110224, 40471, 12100, 9901, and 2601, respectively. Although the scalability of Ex-*µ*-PBIBD scheme is not the best among the above schemes, according to the data in Figure 4, we achieve that the key ring size in Ex*-µ*-PBIBD scheme can enough support the corresponding network size in practical WSNs. 

### 6.2. Key Connectivity

In sBIBD scheme with parameters (*q*^2^ + *q* + 1, *q* + 1, 1), the probability of key shared between each pair of nodes was always 1. Thus, direct key connectivity of BIBD scheme is 1.

In RD scheme with parameters (*q*^2^ + *q* + 1, (*q*^2^ + *q* + 1)(*q* + 1), *q*(*q* + 1), *q*, 1), the probability that any pair of blocks come from same class was given by

(17) $$Q_{SC}=\frac{q^{2}+q-1}{(q^{2}+q)(q^{2}+q+1)-1}$$

and the probability of the pair of blocks shared one or more points was computed as

(18) $$P_{SC}=\frac{q^{2}}{q^{2}+q}.$$

The probability that any pair of blocks come from different classes was given by

(19) $$Q_{DC}=\frac{{\left(q^{2}+q\right)}^{2}}{\left(q^{2}+q+1)((q^{2}+q)(q^{2}+q+1)-1\right)}$$

and the probability that any pair of blocks shared one or more points was computed as

(20) $$P_{DC}=\frac{q^{4}+q-1}{{\left(q^{2}+q\right)}^{2}}.$$

The formula for *Q_SC_*, *P_SC_*, *Q_DC_* and *P_DC_* were given in Ref. [30]. Then key connectivity of RD scheme was expressed as

$${Con}_{RD}=Q_{SC}*P_{SC}+Q_{DC}*P_{DC}$$

where *Q_SC_*, *P_SC_*, *Q_DC_* and *P_DC_* could be computed using Equations (17)–(20).

The key connectivity of Linear TD scheme was estimated as follows:

(21) $${Con}_{TD}=\frac{k}{q+1}$$

**Figure 5:**
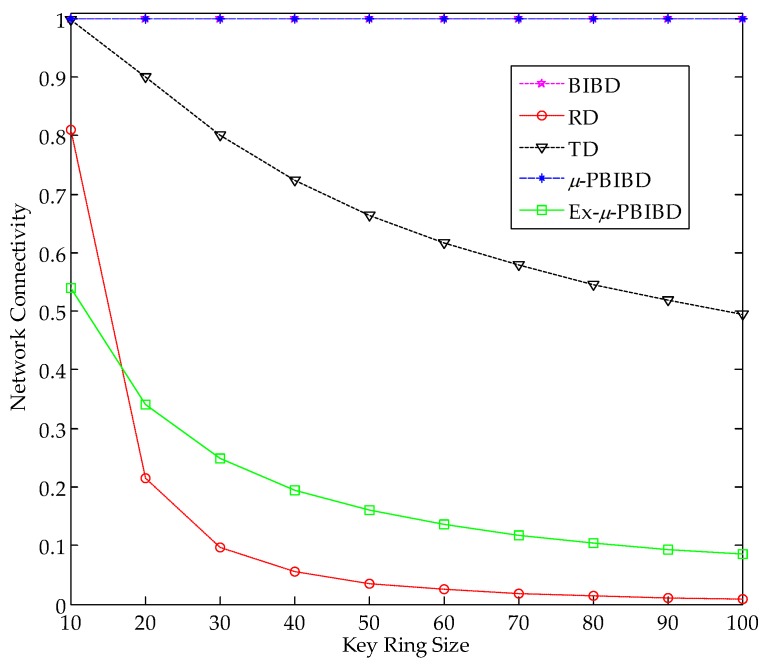
Comparison of key connectivity of different schemes at the key ring size.

Figure 5 shows key connectivity of the four combinatorial schemes. Any pair of nodes in sBIBD scheme and *µ*-sPBIBD scheme have at least one common key. Thus the two schemes have complete connectivity property. The connectivity of Linear TD scheme was determined by parameters *k* and *q*. In order to compare with the connectivity of Linear TD scheme, the network scale of TD scheme should be the same as that of Ex-*µ*-PBIBD. Figure 5 shows that at equal key ring size, Ex-*µ*-sPBIBD scheme has better connectivity than RD scheme when key ring size is more than 13. While it has worse connectivity than TD scheme. We can find that, as key ring size increases, direct connectivity of the proposed scheme decreases in Figure 5. This is due to fact that the probability of key-share tends to *O*(1/*k*) when *k* tends to infinity.

### 6.3. Network Resilience

In this subsection, we discuss network resilience of the five schemes. The network resilience of the sBIBD scheme [32] was calculated as

(22) $$Res_{\mathrm{BIBD}}(x)=1-\frac{\left(\begin{array}{c} q^{2}\\ x \end{array}\right)}{\left(\begin{array}{c} q^{2}+q+1\\ x \end{array}\right)}$$

where *x* represented the number of captured nodes. 

In RD scheme, the network resilience [30] was given by

(23) $$Res_{\mathrm{RD}}(x)=\sum_{j=1}^{q^{2}+q+1}\frac{\left(\begin{array}{c} q(q+1)\\ 2 \end{array}\right)}{\left(\begin{array}{c} \left(q^{2}+q+1)(q+1\right)\\ 2 \end{array}\right)}\left(1-\frac{\left(\begin{array}{c} \left(q+1)(q^{2}+1\right)\\ x \end{array}\right)}{\left(\begin{array}{c} \left(q^{2}+q+1)(q+1\right)\\ x \end{array}\right)}\right)$$

where *x* was the number of captured nodes.

The network resilience of TD scheme in Reference [23] was computed using the following equation:

(24) $$Res_{\mathrm{TD}}(x)=1-{\left(1-\frac{q-2}{q^{2}-2}\right)}^{x}.$$

**Figure 6:**
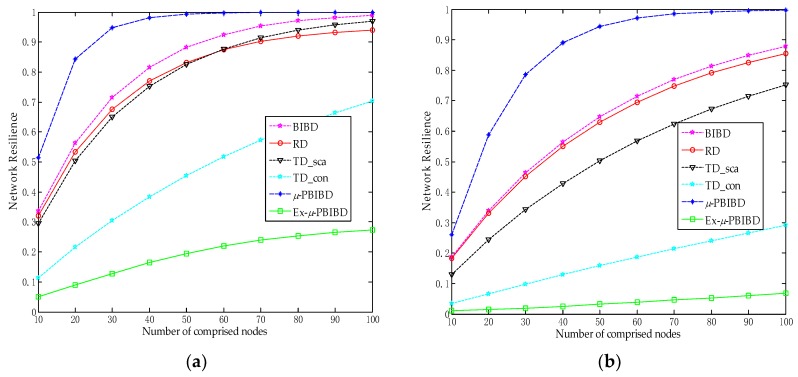
Comparison of resilience of different schemes at the same key ring size. In this figure, resilience is probability of compromised links between two fixed non-compromised nodes versus number of compromise nodes. TD_sca and TD_con are two cases of TD scheme which have the same scalability and connectivity as those of Ex-*µ*-PBIBD, respectively. (**a**) *k* = 24; (**b**) *k* = 48.

In Figure 6, we compare the network resilience of the five schemes at equal number of captured nodes for *k* = 24 and *k* = 48, respectively. In order to compare the performance of TD scheme in a similar setting, we consider two cases of TD schemes which have the same scalability and connectivity as those of our scheme, respectively. According to Figure 6, we can find that Ex-*µ*-sPBIBD scheme provides the best network resilience against compromised nodes in the five schemes. The figures reflect the fact that the network resilience of Ex-*µ*-PBIBD scheme hardly substantially declines, as the number of compromised node increases. Comparing Figure 6a with Figure 6b, the higher *k* is, the better the network resilience is in the case of the same number of captured nodes. That is because the session key between nodes is constructed by shared-keys of key rings between the two nodes. Then more nodes are needed to capture along with the increase of key ring size. 

### 6.4. Additional Analysis

In Ex-*µ*-sPBIBD scheme, connectivity, scalability and resilience are determined by size of key ring (denoted by *k*). Thus, choosing the proper parameter *k* could achieve a trade-off between connectivity and resiliency. Comparing with TD scheme, we should normalize by fixing the size of key ring, *k*, and key connectivity, *Con*. Firstly, we computer connectivity of Ex-*µ*-PBIBD scheme using Equation (3). Next, fixing the size of key ring and the key connectivity, we obtain resilience of TD scheme from Equations (21) and (24) and scalability from Table 3. In Table 4, the parameter choices of schemes are summarized. Then we list the maximum network size (denoted by *M*) and resilience *Res*(*x*) of two schemes. We could select the value of *k* according to requirement of practical WSN.

## 7. Conclusions

In this work, we defined a new combinatorial design, termed “*µ*-PBIBD” and constructed a 2-D *µ*-sPBIBD. We proposed a basic mapping from 2-D *µ*-sPBIBD to KPD which could achieve complete key connectivity and a poor network resilience. To enhance network resilience, we extended a set of keys *V* from 2-D space to 3-D space and proposed an extended 3-D Ex-*µ*-sPBIBD KPD scheme with better network scalability and high network resilience. The theoretical analysis and performance comparison with the existing schemes show that KPD scheme based on Ex-*µ*-sPBIBD increases the network scalability and provides the better network resilience.

## Figures and Tables

**Table 1:** Mapping from 2-D *µ*-sPBIBD to key pre-distribution (KPD).

*µ*-sPBIBD	KPD	Parameter	Value of Parameter
Basic set (point set)	Key pool	*V*	$\left\{(a,b)|(a,b)\in Z_{m}\times Z_{n}\right\}$
Basic set size	key pool size	*v*	*mn*
Block	Key ring	$B_{a,b}$	$\begin{array}{r} \{(i,b),(a,j)|1\leq i\leq m,i\neq a;\\ 1\leq j\leq n,j\neq b\} \end{array}$
Number of blocks	Number of key rings	*d*	*mn*
Block size	Key ring size	*k*	*m* + *n* − 2
Number of common points between two blocks	Number of shared keys between two nodes	$\lambda_{1},\ldots ,\lambda_{\mu }$	2, *m* − 2, *n* − 2

**Table 2:** Mapping from 3-D Ex-*µ*-sPBIBD to KPD.

Ex-*µ*-sPBIBD	KPD	Parameter	Value of Parameter
Basic set	Key pool	*V*	$\left\{(a,b,c)|(a,b,c)\in Z_{q}\times Z_{q}\times Z_{q}\right\}$
Basic set size	Key pool size	*v*	$q^{3}$
Block	Key ring	*B_a,b,c_*	$\begin{array}{l} \{(i,b,c),(a,j,c),(a,b,l)|1\leq i\leq q,\\ i\neq a;1\leq j\leq q,j\neq b;1\leq l\leq q,l\neq c\} \end{array}$
Number of blocks	Number of key rings	*d*	$q^{3}$
Block size	Key ring size	*k*	3q−3
Number of common points between blocks	Number of shared-key between nodes	$\lambda_{1},\cdots ,\lambda_{\mu }$	0,2,q−2

**Table 3:** Parameters of BIBD, RD, TD, 2-D *µ*-PBIBD and 3-DEx-*µ*-PBIBD.

Combinatorial Design	Key Pool Size	Number of Key Rings	Key Ring Size
BIBD [3]	*q*^2^ + *q* + 1	*q*^2^ + *q* + 1	*q* + 1
RD [30]	*q*^2^ + *q* + 1	(*q*^2^ + *q* + 1)(*q* + 1)	*q*
Linear TD [20]	*kq*	*q* ^2^	*k*
2-D PBIBD	*q* ^2^	*q* ^2^	2*q* − 2
3-D EX-PBIBD	*q* ^3^	*q* ^3^	3*q* − 3

**Table 4:** Performance of schemes for values of *k* and *Con* fixed.

Parameter	Linear TD	Ex-PBIBD
*k* = 24 *Con* = 0.3	*M* = 6241 *Res*(40) = 0.3915 *Res*(80) = 0.6297 *Res*(100) = 0.7112	*M* = 729 *Res*(40) = 0.1642 *Res*(80) = 0.2533 *Res*(100) = 0.2728
*k* = 36 *Con* = 0.213	*M* = 28224 *Res*(40) = 0.2102 *Res*(80) = 0.3762 *Res*(100) = 0.4456	*M* = 2179 *Res*(40) = 0.0587 *Res*(80) = 0.1131 *Res*(100) = 0.1390
*k* = 48 *Con* = 0.166	*M* = 82944 *Res*(40) = 0.1290 *Res*(80) = 0.2414 *Res*(100) = 0.2921	*M* = 4913 *Res*(40) = 0.0251 *Res*(80) = 0.0533 *Res*(100) = 0.0677

## Abbreviations

*V*	The basic set
*p_i_*	The *i* point of *V*
*v*	The number of points in *V*
*B*	The block design of *V*
$B_{i}$	The *i* block in *B*
*d*	The number of blocks in *B*
*k*	The number of points in a block (i.e., key ring size)
*r*	The number of blocks in which a point is contain
λ	The number of blocks in which each pair of elements exist
B(v,d,r,k,λ)	Balanced incomplete block design with parameter *v*, *d*, *r*, *k*,λ
*q*	The order of finite projective plane corresponding to *sB*(*q*^2^ + *q* + 1, *q* + 1, 1)
*G*	A partition of *V*
μ	The number of cases on the number of blocks in which each pair of points exist
*M*	the number of senor nodes in WSNs
(*a*, *b*)	The point in *V* in 2-D *µ*-PBIBD
*m*, *n*	The number of row and column when *V* is viewed as 2-D space
$K_{i,j}$	The session key between nodes N*i* and N*j*
*Res*(*x*)	The network resilience when an attacker captures *x* nodes
*pro*_1_	The probability that two blocks share 2 keys
*pro*_2_	The probability that two blocks share *q* − 2 keys
(*a*, *b*, *c*)	The point in *V* in 3-D *µ*-PBIBD
*Con*	The probability that two blocks have shared-key in Ex-*µ*-sPBIBD
N*i*	The *i* node in WSNs
